# Supplementation with a Mango Leaf Extract (Zynamite^®^) in Combination with Quercetin Attenuates Muscle Damage and Pain and Accelerates Recovery after Strenuous Damaging Exercise

**DOI:** 10.3390/nu12030614

**Published:** 2020-02-26

**Authors:** Marcos Martin-Rincon, Miriam Gelabert-Rebato, Victor Galvan-Alvarez, Angel Gallego-Selles, Miriam Martinez-Canton, Laura Lopez-Rios, Julia C. Wiebe, Saul Martin-Rodriguez, Rafael Arteaga-Ortiz, Cecilia Dorado, Sergio Perez-Regalado, Alfredo Santana, David Morales-Alamo, Jose A L Calbet

**Affiliations:** 1 Department of Physical Education and Research Institute of Biomedical and Health Sciences (IUIBS), University of Las Palmas de Gran Canaria, Campus Universitario de Tafira s/n, 35017 Las Palmas de Gran Canaria, Spain; marcos.martinrincon@gmail.com (M.M.-R.); miriamgela@hotmail.com (M.G.-R.); victor_galvan@hotmail.es (V.G.-A.); angelgallegoselles@hotmail.com (A.G.-S.); martinezcantonmiriam@gmail.com (M.M.-C.); saulmrguez@gmail.com (S.M.-R.); rafael.arteaga@ulpgc.es (R.A.-O.); cdoradogarcia@gmail.com (C.D.); serperel284@gmail.com (S.P.-R.); consultageneticasantana@gmail.com (A.S.); moralesalamo.d@gmail.com (D.M.-A.); 2 Nektium Pharma, Agüimes, 35118 Las Palmas de Gran Canaria, Spain; llopez@nektium.com (L.L.-R.); jwiebe@nektium.com (J.C.W.); 3 Complejo Hospitalario Universitario Insular-Materno Infantil de Las Palmas de Gran Canaria, Clinical Genetics Unit, 35016 Las Palmas de Gran Canaria, Spain; 4 Department of Physical Performance, Norwegian School of Sport Sciences, 0806 Oslo, Norway

**Keywords:** ergogenic aids, polyphenols, antioxidant supplementation, eccentric exercise, DOMS, EIMD, sports nutrition, inflammation, human subjects, muscle function

## Abstract

Prolonged or unusual exercise may cause exercise-induced muscle damage (EIMD). To test whether Zynamite^®^, a mango leaf extract rich in the natural polyphenol mangiferin, administered in combination with quercetin facilitates recovery after EIMD, 24 women and 33 men were randomly assigned to two treatment groups matched by sex and 5 km running performance, and ran a 10 km race followed by 100 drop jumps to elicit EIMD. One hour before the competition, and every 8 h thereafter for 24 h, they ingested placebo (728 mg of maltodextrin) or 140 mg of Zynamite^®^ combined with 140 mg of quercetin (double-blind). Although competition times were similar, polyphenol supplementation attenuated the muscle pain felt after the competition (6.8 ± 1.5 and 5.7 ± 2.2 a.u., *p* = 0.035) and the loss of jumping performance (9.4 ± 11.5 and 3.9 ± 5.2%, *p* = 0.036; *p* = 0.034) and mechanical impulse (*p* = 0.038) 24 h later. The polyphenols attenuated the increase of serum myoglobin and alanine aminotransferase in men, but not in women (interaction *p* < 0.05). In conclusion, a single dose of 140 mg Zynamite^®^ combined with 140 mg of quercetin, administered one hour before competition, followed by three additional doses every eight hours, attenuates muscle pain and damage, and accelerates the recovery of muscle performance.

## 1. Introduction

Prolonged or unusual exercise, particularly when involving eccentric muscle contractions, may cause muscle damage [1]. Exercise-induced muscle damage (EIMD) is characterized by muscle soreness, structural disruption, and local inflammation, and it is accompanied by a temporary reduction of muscle force and exercise performance [2]. Although the administration of antioxidants to prevent muscle damage is controversial [3,4,5], some recent studies indicate that ingestion of polyphenol-rich extracts for at least three days before exercise and during the following hours/days may improve recovery [6]. Zynamite^®^, a mango leaf extract rich in the natural polyphenol mangiferin, and quercetin have antioxidant and anti-inflammatory properties [7,8,9,10], which may prevent EIMD and facilitate recovery [11]. Nevertheless, these two polyphenols have not been studied in combination and the effect of quercetin has been only assessed after chronic administration and with large daily doses (∼1000 mg), which could impair exercise performance or blunt part of the adaptations to training [12]. 

In the following hours and days after EIMD, the range of motion is reduced, and some swelling appears in the affected limbs [13]. The risk and magnitude of EIMD is exacerbated when muscle contractions are performed at longer muscle length, faster angular velocity, and with higher forces [2]. Although a mechanical disruption of muscle fibers is thought to be the main mechanism starting EIMD, this is followed by a mild inflammatory response, in which reactive oxygen species (ROS) are involved [14,15]. However, administration of N-acetylcysteine (a thiol-base antioxidant) may be counterproductive, as it has been associated with lower recovery of strength eight days after EIMD [16]. 

It has been shown that Zynamite^®^, a mango leaf extract rich in the natural polyphenol mangiferin, enhances sprint exercise performance when given in combination with luteolin or quercetin [17,18]. Furthermore, both polyphenolic combinations improved the response of human skeletal muscle to ischemia-reperfusion [17,18,19]. Mangiferin (2-bD-glucopyranosyl-1,3,6,7-tetrahydroxyxanthone) is a xanthone abundant in the leaf and pulp of mangoes, which is also present in other edible vegetables [20]. Mangiferin has iron-chelating properties, free-radical scavenging capacity and inhibits some of the oxidases involved in oxidative stress and inflammation [7,9,21,22,23,24]. For these reasons, a natural extract rich in mangiferin may mitigate or prevent EIMD. 

Quercetin is a flavonoid polyphenol that can improve performance during prolonged exercise [25] and sprint exercise when given in combination with Zynamite^®^ [17]. Quercetin can be found in elderberries, onions, cranberries, kale, sophora japonica, apple and many other vegetables, including mangoes. Analogous to mangiferin, quercetin protects against the injury caused by ischemia-reperfusion [26]. Quercetin inhibits xanthine oxidase (XO) and nicotinamide adenine dinucleotide phosphate-oxidase (NADPH oxidase) [27,28]. Thus, conjointly mangiferin and quercetin may counteract, even at low doses, some of the biochemical processes causing EIMD. 

Therefore, the primary aim of this study was to determine whether Zynamite^®^ administered in combination with a small amount of quercetin, which should not interfere with muscle performance, facilitates recovery after repeated damaging exercise. A secondary aim was to determine whether this polyphenol combination attenuates exercise-induced muscle damage and pain.

## 2. Materials and Methods 

### 2.1. Subjects

The students of Sports Sciences (third year, total population *N* = 114) were invited to take part in this research. Fifty-seven volunteered to participate in the study (24 women and 33 men), but only 48 finished the competition and completed the follow-up assessments (18 women and 30 men) (Table 1). Subjects were informed about the inclusion criteria, risks, and benefits of participation and signed a written consent before participation. The investigation was conducted according to the Helsinki Declaration, after approval by the Ethical Committee of the University of Las Palmas de Gran Canaria (CEIH-2019-02). 

The inclusion criteria for participation were: age from 18 to 45 years old; without chronic diseases or recent surgery; non-smoker; normal resting electrocardiogram; body mass index below 30 and above 18; no history of disease requiring medical treatments lasting more than 15 days during the preceding 6 months; no medical contraindications to exercise testing and lack of food allergies. All volunteers applying met the inclusion criteria, except one girl having asthma, which was excluded. All subjects were physically active and exercised regularly.

Subjects were requested to avoid strenuous exercise 48 h preceding all laboratory tests and to refrain from caffeinated, carbonated and alcohol-containing beverages during the 24 h preceding the pre-tests and 48 h prior to the main experiment with supplementation. The subjects were asked to abstain from the consumption of drugs, medications, dietary supplements and the usage of any putative recovery treatments along the whole duration of the study. 

A sample size between 20 and 28 participants was required to provide adequate power to detect an improvement between 5% and 6% in running and jumping performance (α = 0.05, β = 0.80; G*Power v 3.1.9.2). Fifty-seven subjects were recruited to increase the power of our study and account for potential dropouts and missing values. The final sample was reduced to 48 subjects because nine volunteers could not finalize the running race, due to injuries or exhaustion.

### 2.2. General Overview

Subjects first reported to the laboratory for body composition assessment and familiarization with exercise testing. In subsequent days, the pre-tests were carried out to determine their maximal oxygen uptake (VO_2_max) and vertical jump performance. At least one week after the pre-tests, they performed a 5 km running competition on a standard 400 m running track at the university stadium. Subjects competed in small groups matched by performance (5–10 subjects). At least two weeks after the 5 km run, the final competition (main experiment with supplementation) was carried out. All participants had monetary rewards depending on the performance achieved, resulting in a highly competitive environment. The competition consisted of a 10 km race, in the same 400 m track. Two days before the 10 km race, a resting 12 mL blood sample was obtained after a 12-h overnight fast. The 10 km running competition was followed by 100 drop jumps to elicit additional muscle damage. One hour before the 10 km race subjects were administered a placebo or Zynamite^®^ combined with quercetin supplement, as explained below. Following the 10 km race, the volunteers ingested the supplementation dose assigned for three more times, every eight hours until the next day in the morning, when a second 12 mL blood sample was obtained in similar fasting conditions and their vertical jumping performance re-assessed. Five women on the nine assigned to the placebo group and three on the nine assigned to the polyphenol group were taking oral contraceptives.

### 2.3. Pre-Tests and Familiarization

Body composition was determined by dual-energy x-ray absorptiometry (Lunar iDXA, GE Healthcare, Wisconsin; USA) as described elsewhere [29]. Subjects attended two familiarization visits during which an incremental exercise to exhaustion and an all-out sprint were performed. After familiarization, subjects reported to the laboratory to complete different tests on separate days. First, their VO_2_max, maximal heart rate (HRmax) and maximal power output (Wmax) were determined (F_I_O_2_: 0.21, P_I_O_2_: 143 mmHg) with an incremental exercise test to exhaustion with verification [30]. The incremental exercise test started with three min at 20 W, followed by 15 W and 20 W increases every three minutes in women and men, respectively, until the respiratory exchange ratio (RER) was ≥ 1.0. After completion of the intensity with a RER ≥ 1.0, the intensity was increased by 10 and 15 W min^−1^ (women and men, respectively) until volitional exhaustion. The intensity attained at exhaustion was taken at the maximal power output of the incremental exercise test (Wmax). At exhaustion, the ergometer was unloaded, and subjects remained seated on the cycle ergometer pedaling at low cadence (30–40 rpm) for 3 min. After that, the verification test started at Wmax + 5 W for 1 min, followed by increases of 4 and 5 W (women and men, respectively) every 20 s until exhaustion. During all pre-tests, subjects were requested to maintain a pedaling rate close to 80 rpm, while the cycle ergometer (Excalibur Sport 925900, Lode, Groningen, The Netherlands) was set in an rpm-independent mode. 

### 2.4. Main Experiments and Supplement administration

The main competition consisted on a 10 km race run in the same standard 400 m track used for the 5 km run. Participants were randomly assigned to a placebo or polyphenols group. Both groups were matched by running times in the preceding 5 km race, and well as by sex. To elicit EIMD, subjects participated in a 10 km running competition on the athletic track at the university stadium. Race times were registered by an electronic race chip-timing system (Top Time Eventos, Las Palmas de Gran Canaria, Spain) with the runners’ chest-wearing a small lightweight-chip and a number that uniquely identified them when crossing the timing mat at the finish line. Prior to the race, all subjects were instructed to perform their best and that financial incentives would be provided based on their individual performance and compared with other subjects with similar performance in the 5 km race. Verbal encouragement was provided throughout the competition. At the end of the race, capillary blood lactate concentration (earlobe) was assessed at 30–60 s after arrival, followed by a 30–minutes recovery period. During the race and the recovery period, exclusively plain water was provided to drink ad libitum. Then, they performed 100 drop jumps from a 59 cm step in height, consisting of 5 sets of 20 repetitions with a 10-s interval between jumps and interspaced by a 2-min recovery period between sets. Upon landing, the downward movement was stopped allowing the knees to bend up to ∼90°, which was immediately followed by a maximal vertical jump and again at landing stopping at ∼90° of knee flexion. Preceding the drop jumps, all subjects were demonstrated the required technique, and coaching and verbal encouragement were provided during the protocol to ensure that adequate technique and maximal effort were maintained. Two days before and 24 h after the competition, their counter-movement jump performance was assessed on a force platform. During the execution of the counter-movement jumps, the subjects were instructed to keep their hands on the hips and to try to minimize lateral and horizontal displacements. 

To compete during the race, subjects were divided in two groups according to their performance levels. The first group reported to the running track at 7.30 A.M. and the second at 9.30 A.M, having ended their breakfast at least 1 h prior to the scheduled time. Participants were instructed to have a light pre-competition breakfast with their habitual composition consisting in 4–5 kcal kg^−1^ and all subjects thoroughly recorded their breakfast and the last dinner. All subjects were requested not to drink caffeine-containing beverages, taurine or alcohol the 48 h preceding the tests and the competitions. Only mild exercise lasting for no more than 30 min was permitted during the two days preceding the tests and competitions. One hour before the start of the 10 km race, subjects ingested the treatment assigned. The placebo was delivered in the form of two capsules, each containing 364 mg of maltodextrin. The polyphenols were also provided in two capsules each containing 70 mg of Zynamite^®^ (*Mangifera indica* leaf extract, standardized to 60% mangiferin) combined with 70 mg of quercetin in the form of 140 mg of *Sophora japonica* extract standardized to 50% quercetin, and 153 mg of maltodextrin. Three additional doses were ingested every 8 h after the race at lunch, dinner time and in the next morning before the vertical jump test, to a total of 420 mg of Zynamite^®^ combined with 420 mg of quercetin. Both treatments (polyphenols and placebo) were orally administered in opaque and non-distinguishable methylcellulose capsules ingested with 300 mL of water.

### 2.5. Assessment of Pain and Effectiveness of Concealment.

Before, immediately after the 10 km race and 24 h later, subjects were requested to rate the level of pain in their thighs with a visual numerical rating scale from 0 to 10, being 10 the highest muscle pain ever experienced during or after exercise. Participants were inquired while standing with hands on hips and during knee flexion while performing a 90° squat movement. At similar time points, subjects reported their rate of perceived exertion (10-point Borg scale) [31]. All subjects were fully accustomed to rate their level of leg pain and fatigue with the same rating scales due to previous participations in research studies in our laboratory. During the first minute after the race and 24-h later, subjects were asked about the kind of supplement they suspected they had received to check on the effectiveness of concealment.

### 2.6. Vertical Jump Performance 

The forces generated during vertical jumps were measured with a force plate (Kistler, Winterthur, Switzerland), as reported elsewhere [32]. Counter-movement jump (CMJ) performance was tested pre and 24-h post-main experiments, as previously described [33]. Subjects were first familiarized with the correct technique. The execution began from an erect standing position with feet shoulder apart and, when prompted, rapidly descended to reach about 90° of knee flexion and jumped vertically with maximal force. They were aware that the jumps had to be executed explosively to achieve maximum height. During the execution, subjects were instructed to keep their hands on the hips, try to minimize lateral and horizontal displacements, keep their knees straight during the flight phase of the jump and to land in an upright position. On testing days, after a standardized warm-up, participants performed 5 maximal CMJ trials interspaced by 1-min passive rest periods. The vertical velocity at take-off (VT) was determined from the integration of the force applied during the positive phase of the vertical jump [34]. The average jumping height and mechanical impulse developed were used in the subsequent statistical analysis. 

### 2.7. Blood Sampling and Assessment of Biomarkers of Muscle Damage

After a 12-h overnight fast, blood venous samples from an antecubital vein were obtained at baseline and 24-h post-exercise via phlebotomy by a medical doctor. From the total twelve milliliters of blood drawn, 6 mL were placed into serum vacutainer tubes (1 × 8.5 mL) with coagulation enhancer and splitting gel (Ref: 10061, Vacutest Kima, Piove di Sacco, Italy). After ~30 min at room temperature to allow for clotting, blood samples were centrifuged at 2000× g at 4 °C for 10 min. The serum supernatant was aspirated into a series of aliquots and immediately stored at −80 °C and thawed only once before analysis. 

The serum concentration of myoglobin was measured with a chemiluminescent immunoassay (Access Myoglobin, Cat. No. 973243, Beckman Coulter, Brea, California, USA) exhibiting a sensitivity of < 0.1 ng mL^−1^. The intra- and inter-assay coefficients of variation for this assessment were 1.9% and 3.0%, respectively. The serum high-sensitivity C-reactive protein (hs-CRP) concentration was determined spectrophotometrically using an enzyme-linked immunosorbent assay (ELISA) kit commercially available, which was used according to manufacturer´s instructions (catalogue No.: E-EL-H5134) from Elabscience (Houston, TX, USA) with a sensitivity of 9.38 pg mL^−1^ and an intra-assay coefficient of variation of 5.6%. The enzymatic creatine kinase (CK) activity in serum was measured spectrophotometrically by an enzymatic rate method by assaying the rate of NADPH formation using the hexokinase and glucose-6-phosphate dehydrogenase procedure. The sensitivity was 10 U L^−1^ (153 nkat L^−1^) and the intra- and inter-assay coefficients of variation were 1.2% and 3.1%, respectively.

The serum alanine aminotransferase (ALT) activity was determined spectrophotometrically by an enzymatic rate method, by assaying the rate of oxidation of NADH to NAD in the presence of pyruvate and lactate dehydrogenase. In this case, the sensitivity was 3.1 U L^−1^ (0.05 μkat L^−1^), displaying intra- and inter-assay coefficients of variation of 1.1% and 2.4%, respectively.

### 2.8. Statistical Analysis

The values reported are means ± standard deviations. Variables were checked for normal distribution by using the Shapiro–Wilks test. Variables that were not normally distributed were logarithmically transformed. A repeated-measures ANOVA was used with one within-subjects factor: treatment (with two levels: placebo vs. polyphenols) and with sex as a between-subjects factor. The Mauchly’s test of sphericity was run before the ANOVA, and in case of violation of the sphericity assumption, the degrees of freedom were adjusted according to the Huynh and Feldt test. In addition, the changes in percentage were also calculated and compared using a paired two-tailed *t*-test when the ANOVA interaction was statistically significant. Statistical significance was set at a *p* ≤ 0.05. The statistical analyses were performed using IBM SPSS v.21.0 for Apple Computers (IBM, New York, NY, USA).

## 3. Results

### 3.1. Effects on 10 km Running Time, Blood Lactate, RPE and Leg Pain

Both groups had similar physical characteristics, with almost identical VO_2_max and performance in the 5 km run (Table 2 and Table 3). Polyphenol supplementation had no significant effects on running time in the 10 km race (3392 ± 561 and 3409 ± 479 s, for the placebo and polyphenol group, respectively, *p* = 0.91) (Table 4), even after accounting for the 5 km mark recorded before the competition (*p* = 0.24). The response was similar in men and women (treatment by sex interaction *p* = 0.58). Likewise, the ratio in time between the 10 km and 5 km marks was not altered by the intake of polyphenols (2.10 ± 0.12 and 2.07 ± 0.10, for the placebo and polyphenol group, respectively, *p* = 0.40) (Table 4).

Capillary blood lactate concentration at arrival was 36% higher in men than women (6.1 ± 2.6 and 4.5 ±1.4 mM, respectively, *p* = 0.007), without significant differences between treatment groups (Table 4). The RPE at arrival was 12.6% higher in women than men (8.7 ± 1.0 and 7.7 ± 1.3, respectively, *p* = 0.01), without significant differences between treatment groups (Table 4). 

**Figure 1:**
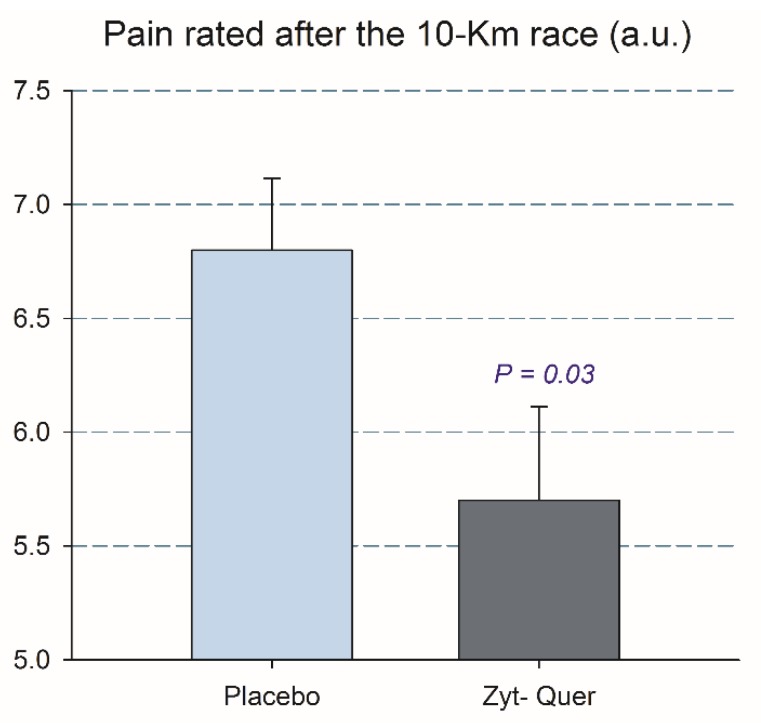
Pain reported at a 90° angle of knee flexion 30 s after arrival from the 10 km race. The error bars represent the standard error of the mean. Placebo, *N* = 23; polyphenols, *N* = 25.

The change in leg pain at a 90° angle of knee flexion elicited by the 10 km competition was lower after the ingestion of polyphenols (6.8 ± 1.5 and 5.7 ± 2.2, respectively, *p* = 0.035) (Figure 1).

### 3.2. Effects on Vertical Jump Performance

**Figure 2:**
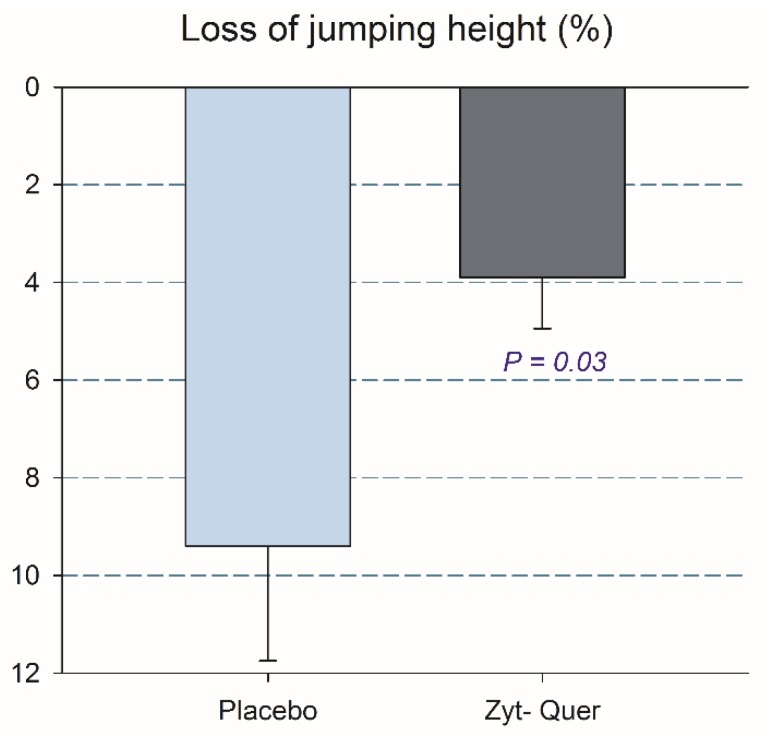
Jumping height loss 24 h after the 10 km race followed by 100 drop jumps. The error bars represent the standard error of the mean. Placebo, *N* = 23; polyphenols, *N* = 25.

**Figure 3:**
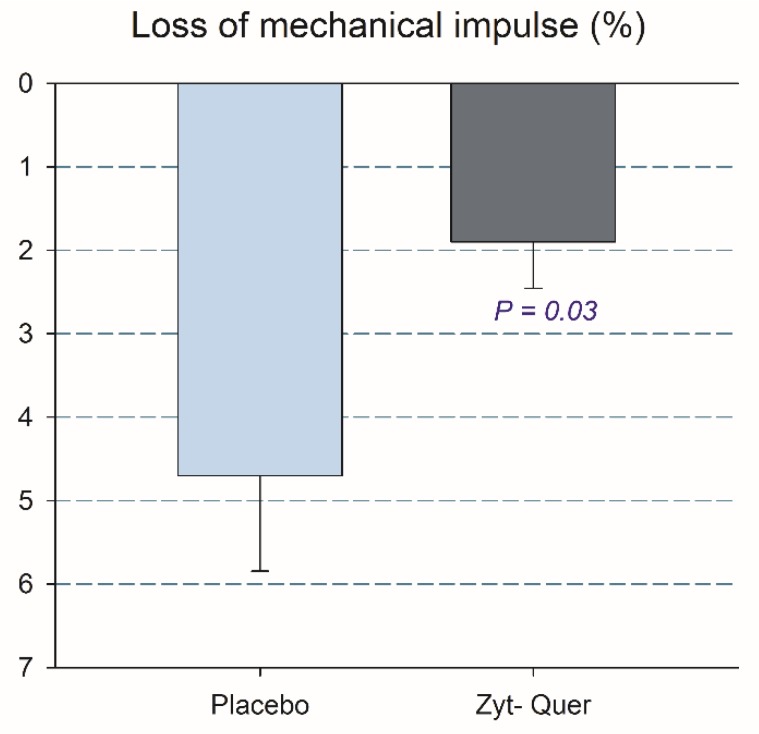
Mechanical impulse lost 24 h after a 10 km race followed by 100 drop jumps. The error bars represent the standard error of the mean. Placebo, *N* = 23; polyphenols, *N* = 25.

Twenty-four hours after the race and the drop jumps, CMJ performance was reduced by 6.9% (*p* < 0.001). The ingestion of polyphenols attenuated the loss of jumping performance (9.4 ± 11.5 and 3.9 ± 5.2%, respectively, *p* = 0.036; time by treatment interaction, *p* = 0.034) (Figure 2). This effect was explained by a lower deterioration of the positive vertical mechanical impulse after the ingestion of polyphenols (4.7 ± 5.6 and 1.9 ± 2.8 %, respectively, *p* = 0.033; time by treatment interaction, *p* = 0.038) (Figure 3).

### 3.3. Biomarkers of Muscle Damage

**Figure 4:**
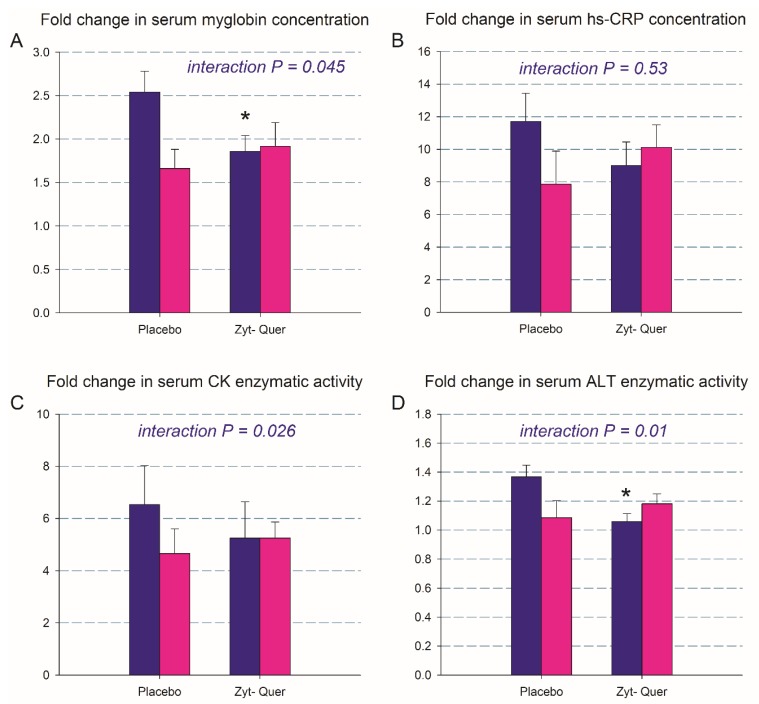
Changes in biomarkers of muscle damage 24 h after a 10 km running competition followed by 100 drop jumps. * *p* < 0.05 compared with placebo. Interaction refers to time by treatment by sex (men: purple; women: pink). The error bars represent the standard error of the mean. Placebo, *N* = 22; polyphenols, *N* = 24.

Myoglobin serum concentration was increased 2-fold 24 h after the exercise protocol (from 24.4 ± 21.2 to 48.3 ± 42.9 ng mL^−1^, *p* < 0.001). The ingestion of polyphenols attenuated the increase of myoglobin in men, but not in women (time by treatment by sex interaction; *p* = 0.045) (Figure 4A). The concentration of hs-CRP in serum was increased 8-fold 24 h after the exercise protocol (0.2 ± 0.2 to 1.9 ± 1.5 mg L^−1^, *p* < 0.001), without a significant effect of polyphenol ingestion (time by treatment interaction, *p* = 0.53; time by treatment by sex interaction, *p* = 0.19) (Figure 4B). CK serum enzymatic activity was increased 3.5-fold 24 h after the exercise protocol (306 ± 623 to 1081 ± 1285 mg L^−1^, *p* < 0.001), without a significant effect of polyphenol ingestion (time by treatment interaction, *p* = 0.95; time by treatment by sex interaction, *p* = 0.26) (Figure 4C). In men, supplementation with polyphenols attenuated the increase of ALT 24 h after the exercise protocol (from 19.3 ± 14.3 to 24.8 ± 15.7 and from 23.3 ± 13.6 to 24.7 ± 16.1 U L^−1^, placebo and polyphenol treatment, respectively, *p* < 0.001; time by treatment interaction, *p* = 0.01) (Figure 4D).

### 3.4. Efficiency of Concealment 

Sixteen of the 23 subjects taking placebo correctly guessed they were on placebo, while only 7 of the 24 subjects on polyphenols guessed that they were taking polyphenols, at the end of the 10 km competition.

## 4. Discussion

This study has shown that a single dose of 140 mg Zynamite^®^ combined with a similar amount of quercetin, taken one hour before exercise eliciting muscle damage, followed by three additional doses every 8 h (420 mg/24 h of each polyphenol during the recovery period), attenuates the pain and the muscle damage elicited by the race and accelerates the recovery of muscle performance. No significant differences in running times were observed between the placebo and the treatment group. 

The protocol here applied induced muscle damage as shown by the degree of pain reported, and the increase of circulating biomarkers of muscle damage and inflammation 24 h after the exercise protocol (myoglobin, CRP, CK and ALT). The increase observed here in CK, CRP and myoglobin is similar to that reported after long distance running [2,35]. Consequently, jumping performance was reduced 24 h after the race and drop jumps, which is one of the best indicators of muscle damage and reduction of muscle functional capacity [36]. 

Exercise-induced muscle damage is initially caused by mechanical disruption of the ultrastructure of muscle [37], which affects several sarcomere proteins [38]. This causes one-half sarcomere nonuniformity and overstretching of sarcomeres beyond filament overlap, reducing the number of myosin-actin cross-bridges and hence, causing a reduction in the capacity to produce force and an overload of the sarcolemma and T-tubule structures [39]. This is followed by the opening of stretch-activated Ca^2+^ channels, membrane ruptures, and excitation-contraction coupling dysfunction. The increase of sarcoplasmic Ca^2+^ may stimulate calpain proteases with loss of contractile proteins prolonging the loss of force [40,41,42]. An intrinsic characteristic of EIMD is the induction of a protective effect when a similar exercise is repeated within the following days after the first damaging exercise (repeated bout effect) [43]. In the present investigation, we took care to measure well the performance levels of our subjects, although using a shorter distance to minimize the repeated bout effect. Despite a potential protective effect from the 5 km run, our protocol elicited EIMD likely due to the longest distance of the main competition, and the execution of 100 drop jumps 30 min after the 10 km race.

The muscle pain is likely triggered by inflammation of the extracellular matrix [36], by neurotrophic factors released by the muscle fibers and satellite cells, as well as by invading polymorphonuclear cells in the following days [39]. This causes a mild inflammatory response in which reactive oxygen species are involved [14,15]. Likely, several mechanisms act conjointly to elicit pain, but the nature may differ in the immediate post-exercise phase from the subacute phase (48–72 h after the exercise bout, which was not assessed here). Despite the hypothesized role of ROS in EIMD [14,15], the administration of N-acetylcysteine (a thiol-base antioxidant) may be counterproductive, as it has been associated with lower recovery of strength eight days after the exercise [16]. The protective effects of polyphenols after prolonged administration may depend more of the stimulation of the endogenous antioxidant systems through Nrf2 and antioxidant response element pathway signaling [44,45,46], rather than on a direct free radical-scavenging effect [2]. In the case of the combination of Zynamite^®^ with quercetin, the antioxidant/anti-inflammatory effect due to the inhibition of the ROS-producing enzymes XO and NADP oxidase may explain the effect observed even though only a single dose was administered before exercise.

In the present study, the administration of Zynamite^®^ combined with quercetin attenuated the muscle pain reported at the end of the race. In agreement, a trend to lower pain during post-exercise ischemia was observed for this polyphenolic combination, although given at larger doses [17]. This analgesic effect could have been mediated by the free radical-scavenging properties of Zynamite^®^ and quercetin since free radicals have been implicated in nociception [47]. In addition, adenosine accumulation due to XO inhibition by Zynamite^®^ and quercetin may have partly attenuated nociception reducing the pain felt by the subjects that received the polyphenol mixture, as suggested by animal experiments with allopurinol [48]. In agreement with our results, analgesic effects counteracting exercise-induced muscle pain have been reported in previous studies with polyphenol supplementation during the days preceding the exercise [6].

In the current investigation, we employed a realistic model of muscle damage, by using normal athletic activities of the lower extremities, which are more resistant to muscle damage than the upper extremities [49,50]. Therefore, not surprisingly, the loss of jumping performance was moderate, but similar to that reported following eccentric leg exercise on isokinetic dynamometers [50]. Interestingly, our polyphenolic combination was associated with faster recovery of jumping performance. In agreement with our findings, a more rapid recovery of muscle performance after eccentric exercise has also been reported for other polyphenol-rich extracts, in a dose-dependent effect [6].

Although EIMD is similar between men and women, the inflammatory response is more marked in men [51,52]. This sex dimorphism has been attributed to the anti-inflammatory and antioxidant properties of estrogens [53,54]. In the women studied here, polyphenols accelerated the recovery of performance and muscle pain, regardless of the intake of oral contraceptives. Five women in the placebo group were taking oral contraceptives, which may have contributed to attenuate the release of biomarkers of muscle damage in the placebo group [54], masking a potentially similar effect by the polyphenols. 

This study has some limitations. The experimental design employed in the present investigation impedes to ascribe the results reported here to additive or synergistic effects of the polyphenol combination. Besides, the sample size might have been small to show significant effects of this polyphenol combination on biomarkers of muscle damage in the female group and, therefore, we cannot rule out a type II error. Although the diet was not standardized and the polyphenol content of the usual diet was not determined, the randomized adscription to each group should have minimized any potential bias due to differences in the diet.

## 5. Conclusions

In summary, a single dose of 140 mg Zynamite^®^ combined with a similar amount of quercetin, taken one hour before exercise eliciting muscle damage, followed by three additional doses every 8 h (420 mg/24 h of each polyphenol during the recovery period), attenuates the pain felt after the exercise, reduces the muscle damage caused by the exercise, and accelerates the recovery of muscle performance. These effects are likely due to the antioxidant and anti-inflammatory properties of the combination of Zynamite^®^ with quercetin. No similar results have been reported previously for such a low dose and short supplementation time when quercetin is ingested alone. 

## Figures and Tables

**Table 1:** Physical characteristics, body composition and VO_2_max.

	Men			Women			*p*
Age (years)	23.1	±	2.5	23.3	±	3.4	0.75
Height (cm)	176.6	±	5.8	165.7	±	5.2	0.000
Weight (kg)	74.7	±	6.8	61.0	±	6.2	0.000
Body fat (%)	18.7	±	4.1	26.9	±	4.5	0.000
Fat body mass (kg)	14.1	±	3.8	16.5	±	4.2	0.04
Lean body mass (kg)	57.4	±	5.3	41.9	±	3.8	0.000
Legs lean mass (kg)	20.2	±	2.1	14.8	±	1.6	0.000
VO_2_max (mL min^−1^)	3246	±	358	2360	±	415	0.000
VO_2_max (mL kg^−1^ min^−1^)	43.6	±	3.8	38.6	±	4.5	0.000
LLM VO_2_max (mL kg^−1^ min^−1^)	161.3	±	15.4	159.4	±	18.1	0.71

LLM: lower extremities lean mass, also legs lean mass. *N* = 18 and 30 for women and men, respectively.

**Table 2:** Physical characteristics, body composition and VO_2_max by treatment group.

	Placebo			Polyphenols			*p*
Age (years)	23.9	±	3.2	22.5	±	2.2	0.08
Height (cm)	172.3	±	8.3	172.6	±	7.2	0.90
Weight (kg)	69.2	±	10.0	69.8	±	9.4	0.85
Body fat (%)	21.0	±	6.7	22.5	±	4.8	0.37
Fat body mass (kg)	14.3	±	4.5	15.6	±	3.7	0.29
Lean body mass (kg)	52.0	±	9.8	51.2	±	8.2	0.77
Legs lean mass (kg)	18.2	±	3.4	18.1	±	3.2	0.91
VO_2_max (mL min^−1^)	2898	±	621	2928	±	539	0.86
VO_2_max (mL kg^−1^ min^−1^)	41.6	±	4.6	41.9	±	4.9	0.83
LLM VO_2_max (mL kg^−1^ min^−1^)	159	±	11	163	±	20	0.40
5-km run (s)	1611	±	212	1642	±	201	0.60

LLM: lower extremities lean mass, also legs lean mass. *N* = 23 and 25 for placebo and polyphenols group, respectively.

**Table 3:** Physical characteristics, body composition, VO_2_max and 5 km running performance.

	Placebo							Polyphenols						
	Men (*N* = 14)			Women (*N* = 9)			*p*	Men (*N* = 16)			Women (*N* = 9)			*p*
Age (years)	23.9	±	2.5	24.0	±	4.3	0.91	22.4	±	2.2	22.7	±	2.4	0.76
Height (cm)	177.4	±	4.8	164.4	±	6.0	0.000	175.9	±	6.5	166.9	±	4.3	0.001
Weight (kg)	74.3	±	8.0	61.3	±	7.4	0.001	74.9	±	6.6	60.7	±	6.2	0.000
Body fat (%)	16.9	±	4.0	27.3	±	4.9	0.000	20.2	±	3.5 *	26.4	±	4.2	0.001
Fat body mass (kg)	12.7	±	3.8	16.9	±	4.4	0.02	15.3	±	3.6	16.2	±	4.2	0.55
Lean body mass (kg)	58.4	±	6.0	42.0	±	4.5	0.000	56.5	±	4.5	41.9	±	3.3	0.000
Legs lean mass (kg)	20.3	±	2.3	15.0	±	1.8	0.000	20.1	±	2.0	14.6	±	1.3	0.000
VO_2_max (mL min^−1^)	3285	±	416	2297	±	334	0.000	3211	±	310	2424	±	495	0.000
VO_2_max (mL kg^−1^ min^−1^)	44.2	±	3.5	37.4	±	2.4	0.00	43.0	±	4.1	39.8	±	5.8	0.12
LLM VO_2_max (mL kg^−1^ min^−1^)	162	±	11	153	±	8	0.06	161	±	19	166	±	23	0.58
5-km run (s)	1509	±	144	1770	±	209	0.002	1558	±	159	1792	±	186	0.003

LLM: lower extremities lean mass, also legs lean mass. *p*-values for the comparison between men and women into each treatment group; * *p* < 0.05 compared with the placebo group.

**Table 4:** Performance and pain after the 10 km race.

	Placebo							Polyphenols						
	Men (*N* = 14)			Women (*N* = 9)			*p*	Men (*N* = 16)			Women (*N* = 9)			*p*
10-km race (s)	3128	±	357	3802	±	590	0.003	3209	±	351	3763	±	487	0.003
Ratio 10km/5Km	2.07	±	0.12	2.14	±	0.10	0.18	2.06	±	0.10	2.10	±	0.10	0.43
Lactate (mM)	5.9	±	2.4	4.8	±	1.2	0.21	6.2	±	2.8	4.1	±	1.6	0.05
RPE Post 10 Km	7.9	±	1.2	8.7	±	1.1	0.11	7.6	±	1.5	8.6	±	0.9	0.06

Ratio 10 km/5 km: the ratio between the performance time in the 10 and 5 km-competitive races; Lactate: capillary blood lactate concentration at the 10 km race arrival; RPE: rate of perceived exertion at arrival. *p*-value for the comparison between men and women into each group. No significant differences were observed between the placebo and the polyphenols group.
